# The Mechanical Properties of Biocompatible Apatite Bone Cement Reinforced with Chemically Activated Carbon Fibers

**DOI:** 10.3390/ma11020192

**Published:** 2018-01-26

**Authors:** Anne V. Boehm, Susanne Meininger, Annemarie Tesch, Uwe Gbureck, Frank A. Müller

**Affiliations:** 1 Otto Schott Institute of Materials Research (OSIM), Friedrich Schiller University Jena, Löbdergraben 32, 07743 Jena, Germany; anne.boehm@uni-jena.de (A.V.B.); annemarie.tesch@uni-jena.de (A.T.); 2 Department for Functional Materials in Medicine and Dentistry (FMZ), University of Würzburg, Pleicherwall 2, 97070 Würzburg, Germany; susanne.meininger@fmz.uni-wuerzburg.de (S.M.); uwe.gbureck@fmz.uni-wuerzburg.de (U.G.)

**Keywords:** calcium phosphate cement, damage tolerant cement, carbon fiber reinforcement, interface control, fiber–matrix interaction

## Abstract

Calcium phosphate cement (CPC) is a well-established bone replacement material in dentistry and orthopedics. CPC mimics the physicochemical properties of natural bone and therefore shows excellent in vivo behavior. However, due to their brittleness, the application of CPC implants is limited to non-load bearing areas. Generally, the fiber-reinforcement of ceramic materials enhances fracture resistance, but simultaneously reduces the strength of the composite. Combining strong C-fiber reinforcement with a hydroxyapatite to form a CPC with a chemical modification of the fiber surface allowed us to adjust the fiber–matrix interface and consequently the fracture behavior. Thus, we could demonstrate enhanced mechanical properties of CPC in terms of bending strength and work of fracture to a strain of 5% (WOF5). Hereby, the strength increased by a factor of four from 9.2 ± 1.7 to 38.4 ± 1.7 MPa. Simultaneously, the WOF5 increased from 0.02 ± 0.004 to 2.0 ± 0.6 kJ∙m^−2^, when utilizing an *aqua regia*/CaCl_2_ pretreatment. The cell proliferation and activity of MG63 osteoblast-like cells as biocompatibility markers were not affected by fiber addition nor by fiber treatment. CPC reinforced with chemically activated C-fibers is a promising bone replacement material for load-bearing applications.

## 1. Introduction

The chemical and crystallographic similarity of hydroxyapatite (HAp) to bone apatite mineral found in mammalian hard tissues has made this material most attractive for replacing human bones and teeth [1,2,3,4]. Calcium phosphate cement (CPC) for bone regeneration applications has been under intense research for more than 30 years now [5]. Numerous formulations have been examined involving tailoring of the degradability, application time, and mechanical properties of CPC [6,7,8]. Commercial products are already in use for non-load bearing applications, like craniofacial or maxillodental surgery [7,9,10]. However, the main problem of CPC is related to its brittleness, resulting in a low fracture toughness [11]. To overcome this problem, reinforced CPC has been investigated using various reinforcement strategies [12,13], with particles or whiskers [14,15], short fibers for e.g., injectable CPC [16,17], and long fibers [18]. In a previous work on different fiber reinforcements, carbon fibers (C-fibers) proved their suitability for enhancing the strength and work of fracture (WOF) of CPC [8]. Xu et al. [18,19] reported on the mechanics of C-fiber reinforced CPC composites. They found that 5.7 vol % of C-fibers with a length of 75 mm in apatite cement resulted in a bending strength and WOF of 59 ± 11 MPa and 6.6 ± 1.2 kJ∙m^−2^, respectively, when compared to pure CPC with a strength of 13 ± 3 MPa and a WOF of 0.04 ± 0.01 kJ∙m^−2^. The main reinforcement mechanism was found to be the pull-out of fibers over long distances, which results from the weak interfacial strength between the matrix and continuous fiber reinforcement.

Although the strengthening and toughening of CPC have already been achieved through the use of C-fibers, to the best of our knowledge, the surface functionalization of the fibers has not yet been considered, and therefore the full potential of this reinforcement strategy is still not exploited. Adjusting the fiber–matrix interface by activating the fibers’ surface alters the wetting and precipitation process and will consequently influence the mechanical properties and the failure mechanism. In the present work, we focus on the mechanical properties of CPC reinforced with C-fibers that were activated by utilizing different oxidation agents, followed by a calcium adhesion process. Since the addition of untreated fibers and particularly the chemical pretreatment of the fibers can have a decisive impact on the cellular behavior of these composites, their biocompatibility was tested with the osteoblast-like cell line MG63.

## 2. Results and Discussion

### 2.1. Fiber Activation

**Figure 1:**
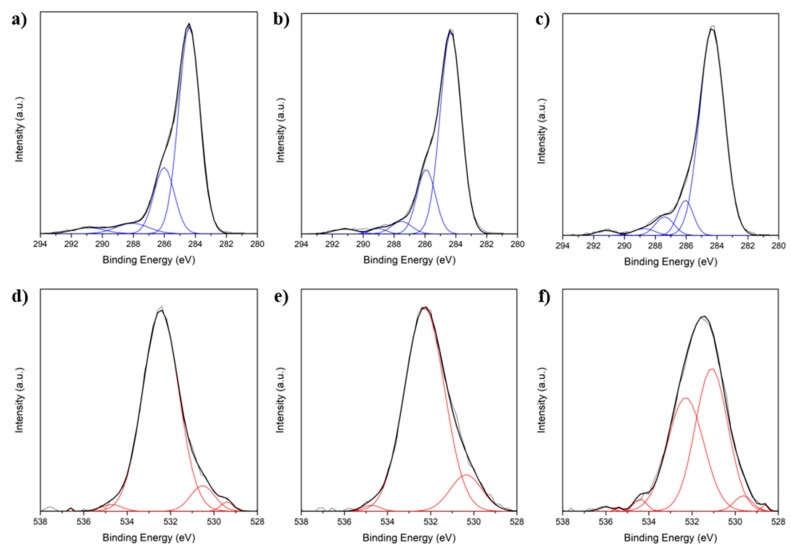
C1s (**a**–**c**) and O1s (**d**–**f**) measured by X-ray photoelectron spectroscopy for (**a**,**d**) untreated; (**b**,**e**) H_2_O_2_-treated; and (**c**,**f**) *aqua regia*/CaCl_2_-treated C-fibers.

Modification of the fibers by hydrogen peroxide (H_2_O_2_) or *aqua regia* and the subsequent calcium adhesion (*aqua regia*/CaCl_2_) was characterized by X-ray photoelectron spectroscopy (XPS) and contact angle measurements, as shown in Figure 1 and Table 1, respectively. Spectra of the C1s and O1s XPS peaks were measured. Due to oxygen adsorption during exposure to air as well as during fiber processing, starting from the precursor polymer polyacrylonitrile (PAN), oxygen is hence present in all fibers, regardless of modification. Beyond oxygen absorption, oxide lattice oxygen was also detected at binding energy (BE) 529–530 eV as a side effect of fiber processing [20].Therefore, the interpretation of the quantity of oxygen or the oxygen to carbon ratio is disputable in this case. However, by analyzing the signals with single-peak fitting (blue curves in C1s spectra, red in O1s spectra), different bond types can be assigned to the binding energies (BEs) and therefore the surface chemistry can be discussed.

The predominant species found in the C1s spectra (Figure 1a–c) of all fiber treatments are aromatic or aliphatic carbon, which are also known for graphite at binding energies (BEs) of 284.4 eV [21]. Additionally, untreated fibers and H_2_O_2_-oxidized fibers evolve signals at BE = 285.9–286.0 eV which can be attributed to hydroxyl groups (–OH) [22]. In the case of the *aqua regia*/CaCl_2_ treatment, the spectra shift to a higher BE with a peak at 288.8 eV occurs, which can be attributed to carboxyl groups (–COOH) [23]. Furthermore, small quantities of adsorbed carbon oxide (CO) or carbon dioxide (CO_2_) were detected for all fibers at a BE of roughly 291 eV [24].

Focusing on the O1s spectra (Figure 1d–f), only small differences between untreated fibers and H_2_O_2_ modification occurred. In those cases, the predominant oxygen species is found at a BE of 532.2–532.5 eV which can be attributed to –OH [22]. In contrast, oxidation using *aqua regia* resulted in a shift of the O1s signal towards lower BE, namely an additional peak at 531.0 eV which can be assigned to carbonyl groups (–CO) [21,22,24]. Furthermore, for all fiber types, but with different relative intensity, carboxyl groups (–COOH) were detected at BEs of 534.2–534.9 eV [21,22,24]. Hereby, the relative intensity, referring to the O1s intensity, is related to the fraction of this group. An increased ratio of –COOH was observed in the case of an *aqua regia*/CaCl_2_ treatment. 

Combining both spectra, several types of oxygen binding can be assigned. In the case of untreated and H_2_O_2_-pretreated fibers, mostly adsorbed oxygen and –OH groups are present, whereas the treatment with *aqua regia* causes the occurrence of –CO and –COOH. Considering that the setting reaction proceeds under basic conditions, a partial dissociation of those groups is possible. Taking into account pK_a_-values of 9–11 for –OH [21,22], only a very minor fraction of –OH groups is deprotonated and hence no calcium adhesion was found in the XPS spectra for untreated and H_2_O_2_-treated fibers. On the contrary, the lower pK_a_ values of ~2–4 for the hydration of carbonyl compounds with hydroxy ions (OH^−^) [23,24] result in significant amounts of deprotonated –OH, which, like deprotonated carboxylic acid groups (COO^−^), have a strong tendency to bind calcium. Therefore, XPS proved calcium adhesion to the *aqua regia*-pretreated C-fibers.

In Table 1 the water contact angles of untreated and chemically modified C-fibers are shown. The fiber oxidation leads to a significant decrease of the contact angles from 71° for untreated fibers to 64° for H_2_O_2_-treated and to 62° for *aqua regia*/CaCl_2_-treated fibers. Reduced contact angles represent enhanced wettability, which was also observed during the handling and manufacturing of CPC composite samples.

### 2.2. Cement Setting

**Figure 2:**
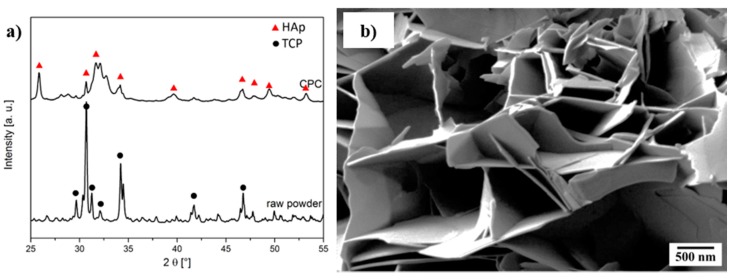
Characterization of (**a**) the phase composition of raw powders and set cement using X-ray diffraction and (**b**) the morphology of the cement matrix by scanning electron microscopy.

**Figure 3:**
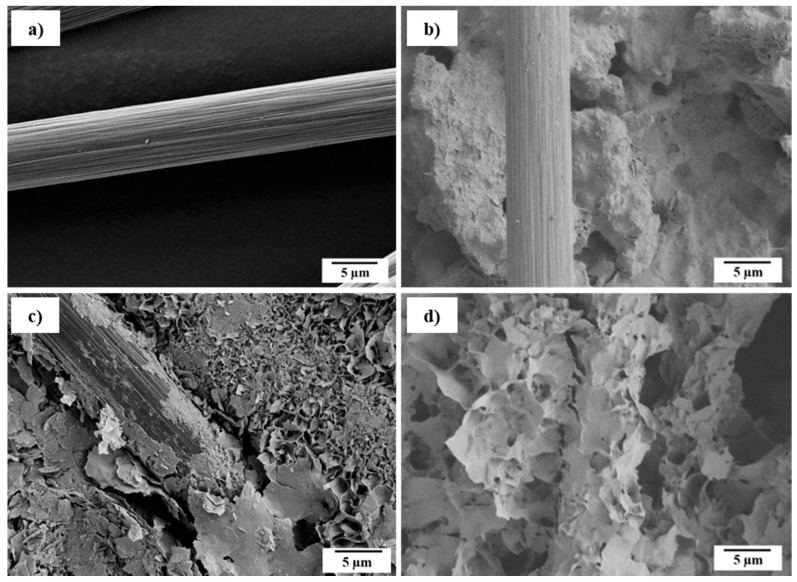
Scanning electron microscopy of (**a**) untreated fibers and in (**b**) cement; as well as (**c**) H_2_O_2_-treated fibers and (**d**) *aqua regia* and CaCl_2_-treated fibers in cement.

The setting reaction from α-tricalcium phosphate (α-TCP) to calcium deficient hydroxy apatite (CDHA) was characterized using X-ray diffraction (XRD) and SEM (Figure 2). Hereby, the plate-shaped precipitation of CDHA was confirmed. The kinetics of the setting reaction were investigated using the Gilmore needle test (Table 1). Both initial and final setting time are reduced significantly by inducing fibers. The reduced initial and final setting times are preferable for medical applications, due to time savings in the surgery process [25]. Especially in the case of the initial setting time, the reduction can be attributed to fibers acting as heterogenous seeds for the precipitation. The strongest reduction was observed in the case of *aqua regia*/CaCl_2_ treatment, where fibers had already bound Ca^2+^ ions. Thus, the first step for apatite nucleation has already occurred and PO_4_^3−^ is directly attracted to such sites. Consequently, the crystal growth is accelerated the most. This effect is also represented by the final setting time, where a shortening by 50% was observed for the composite. Since the cement morphology around the fibers changed, one can assume that the precipitation process varies for different fiber modifications (Figure 3). Whereas untreated fibers show almost no interaction with the CPC matrix (Figure 3b), crystal growth can be observed on H_2_O_2_-treated (Figure 3c) as well as on *aqua regia*/CaCl_2_-treated fibers (Figure 3d). In the latter case the crystals appear bigger than for the H_2_O_2_ treatment, which indicates a faster crystal growth or a slower nucleation rate. It is of value to mention that due to the acidic character of the fiber surface, not only the calcium phosphate nucleation process is affected or altered, but the chemical composition of the precipitate could also differ. Under the given conditions, besides CDHA, either brushite or octacalcium phosphate (OCP) could be precipitated. However, due to the addition of sodium citrate, the precipitation of brushite is inhibited, and therefore its formation seems unlikely [26,27]. On the other hand, the formation of fast growing OCP crystals is possible at high supersaturation on an acidic fiber surface [28]. In conclusion, two explanations for the reduced setting time in the case of an *aqua regia*/CaCl_2_ treatment are possible. Either the setting time is reduced by an accelerated crystal growth due to the presence of calcium binding groups and a calcium pre-saturation on the fiber surface, or the change of precipitation product from CDHA to OCP, which shows faster crystal growth than CDHA may be a factor. However, the formation of OCP is only likely in close proximity to the fibers’ surface, but not in the setting of the CPC matrix that consists of CDHA.

### 2.3. Mechanical Properties

**Figure 4:**
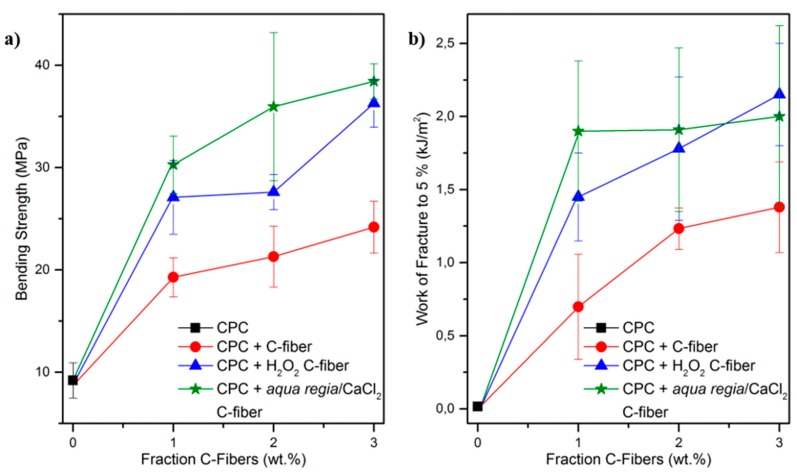
(**a**) Bending strength and (**b**) work of fracture up to 5% strain of C-fiber reinforced calcium phosphate cement (CPC) depending on the fiber fraction for different chemical fiber surface modifications.

**Figure 5:**
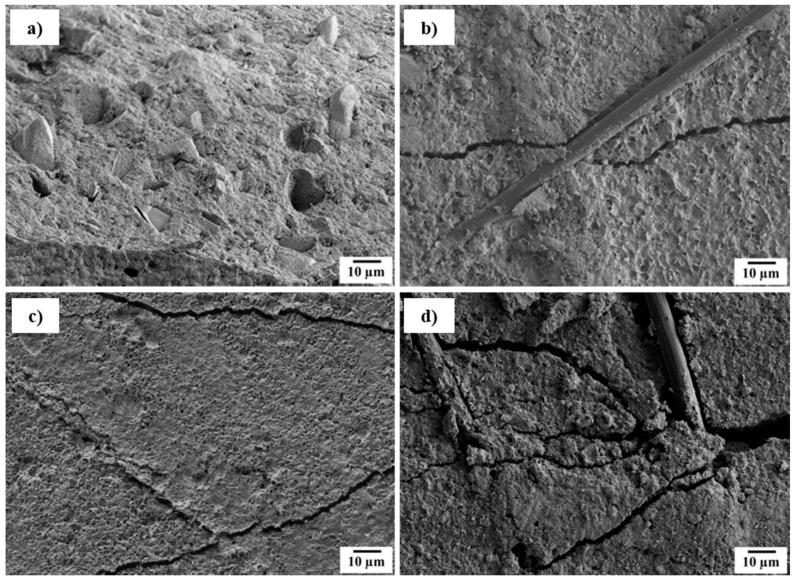
Scanning electron microscopy of (**a**) the fracture plane for pure calcium phosphate cement (CPC), and crack propagation at 1% strain for: (**b**) untreated; (**c**) H_2_O_2_-treated and (**d**) *aqua regia*- and CaCl_2_-treated fiber-reinforced CPC with a fiber content of 1 wt %.

Figure 4 shows the bending strength (Figure 4a) and work of fracture to 5% strain (WOF5, Figure 4b) of CPC reinforced with differently modified C-fibers. Pure cement shows brittle behavior, as illustrated in SEM micrographs of the crack plane (Figure 5a) and a negligible WOF5 of 0.02 kJ⋅m^−2^ (Figure 4b). Unreinforced specimens are stressed until a single crack starts at a critical defect and propagates catastrophically. Only a few deviations from the direct crack path are observed, which are due to unreacted α-TCP particles or the crystallite structure of the matrix itself (Figure 5a). In the case of a C-fiber reinforcement, the addition of fibers leads to a stabilization of the crack opening and therefore steady-state cracking [29]. Composites show significantly increased strength and WOF5, even at the incorporation of only 1 wt % of untreated C-fibers. Strength was doubled, whereas the WOF5 was increased by a factor of 35 from 0.02 to 0.70 kJ∙m^–2^. This effect was already described in literature [12,18,19] and is explained by changes in the force absorption and cracking mechanism due to the incorporation of a second phase. The main effects are crack tilting and twisting, as intensively studied by Faber and Evans [30,31,32]. When incorporating more fibers (2 and 3 wt % C-fibers) the strength further increases, due to a higher fraction of the stronger reinforcement phase. Additionally, the WOF5 increases because the composite benefits from the higher interface ratio between fiber and matrix, leading to higher friction due to additional pull-outs.

However, untreated C-fibers show no chemical interaction with the CPC matrix. Thus, the main energy-consuming effect is related to fiber pull-out. In the case of H_2_O_2_-treated fibers, the slightly higher hydrophilicity, as well as the occurrence of hydroxy groups on the fiber surface, leads to a weak chemical interaction with Ca^2+^ in the cement matrix. Therefore, the force transfer from matrix to fiber is enhanced and, consequently, the bending strength increases in comparison to an untreated fiber reinforcement (Figure 4a). Beyond, weak interfacial forces increase the resistance against fiber pull-out and therefore the energy consumption, resulting in an increased WOF5 (Figure 4b).

In the case of an *aqua regia*/CaCl_2_ pretreatment, the incorporation of fibers with polar carbonyl and carboxyl surface groups causes changes in the cracking mechanism, since these groups already bound Ca^2+^ chemically and show strong interactions with the CPC matrix. Here, the force transfer from matrix to fiber is working most effectively and, therefore, the bending strength in comparison to unmodified fibers is almost doubled even at only 1 wt % fiber content, further increasing with raised fiber fraction. SEM micrographs (Figure 5d) reveal a change in the crack pattern. In comparison to the other treatments (Figure 5b,c), a higher number of cracks with smaller widths are visible. Due to their interfacial properties bridging fibers are able to sustain the total load and transfer the force back into the matrix through interfacial shear. Consequently, this leads to the formation of another crack. This process is repeated several times, resulting in multiple cracking [33]. This force transfer also becomes evident in the crack path. Whereas for untreated and H_2_O_2_-treated fiber composites, cracks were propagating along the fiber–matrix interface, in CPC with *aqua regia*/CaCl_2_-treated C-fibers, cracks were stopped, split, deflected, and propagate through the matrix material instead of only along the interface. This indicates that the resistance against pull-out and delamination is higher, and the error size distribution is augmented. Consequently, more cracks are opened. In the case of WOF5 no significant increase with fiber content is noticeable. In contrast to untreated and H_2_O_2_-treated fibers, the composite with *aqua regia*/CaCl_2_ fibers does not benefit from a higher fiber content and interface ratio. However, a fiber fraction as low as 1 wt % is sufficient to initiate steady-state cracking. This mechanism is more effective in reinforcing and therefore both the strength and WOF5 are the highest after *aqua regia*/CaCl_2_ treatment.

### 2.4. In Vitro Biocompatibility.

**Figure 6:**
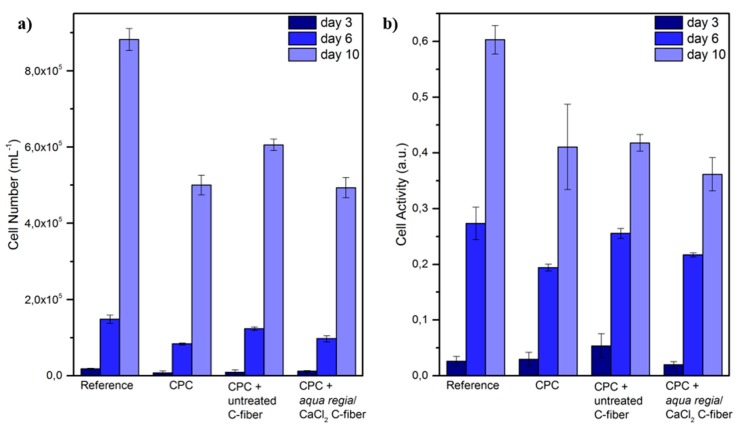
(**a**) Cell number and (**b**) cell activity of osteoblast-like cell line MG63. Cells were cultivated with medium from dissolution tests over 10 days.

An elution test was performed to assess the biocompatibility of the cement and a possible influence of the fiber reinforcement. CPC disks were immersed and dissolved in cell culture medium, which was analyzed with regard to ionic changes and used to culture osteoblast-like cells of the cell line MG63. Cell proliferation was determined by cell detachment and counting (Figure 6a). After 3 days of incubation, the cell number was slightly higher than the seeding concentration for all samples. However, cells proliferated during culture. After 10 days, the cell number was between 60- and 70-fold, and although the reference (fresh media) had an even higher cell number (100-fold), none of the dissolution products of the samples had an inhibiting effect on osteoblast proliferation. The cell activity measured by the reaction with water-soluble tetrazolium (WST) reagent exhibited a similar behavior (Figure 6b). Here, the activity almost doubled every 3–4 days. Again, the metabolic activity of the reference was slightly higher than for the cement samples, but was steadily increasing. In both cases, no difference between the pure CDHA and the fiber-reinforced CDHA could be detected. Also, the fiber treatment had no impact on the cellular behavior. The results had a very low standard deviation indicating that the cellular response to ion release and adsorption was constant and was, therefore, reliable.

**Figure 7:**
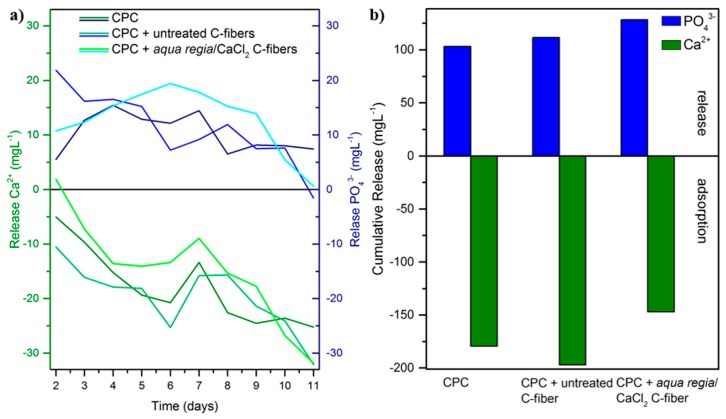
(**a**) Ca^2+^ and PO_4_^3−^ release during dissolution of calcium phosphate cement (CPC) with and without fiber-reinforcement, as well as (**b**) cumulative release and adsorption, respectively, over a dissolution period of 11 days.

Since changes in the ion concentration are the most influential factor on cellular behavior for calcium phosphate-based biomaterials [34,35], the supernatant of dissolving samples was investigated with inductively coupled mass spectrometry (ICP-MS). Both Ca^2+^ and PO_4_^3−^ changes in the culture media were calculated against fresh media for each day (Figure 7a). Independent of fiber addition and fiber treatment, for all samples phosphate ions were released into the media, whereas calcium ions were adsorbed on the sample surface. The PO_4_^3−^ release showed a maximum of around 21 mg⋅L^−1^ and decreased during dissolution to almost no release after 11 days. In contrast to that, the adsorption of Ca^2+^ ions started with a low adsorption and increased steadily to a maximum adsorption of around 33 mg⋅L^−1^ after 11 days. Additionally, the total release over 11 days was summarized, (Figure 7b). Again, there was no significant difference between pure CPC and fiber-reinforced CPC samples. The cumulative PO_4_^3−^ release ranged between 100 and 130 mg⋅L^−1^ and the cumulative Ca^2+^ adsorption was around 150 to 200 mg⋅L^−1^. Although oxidized C-fibers showed a stronger tendency to bind Ca^2+^ ions, increased adsorption from culture media was not observed. On the contrary, over 11 days, *aqua regia*/CaCl_2_-pretreated samples showed the lowest adsorption. Considering the pre-saturation of C-fibers in the additional CaCl_2_ exposure step, this effect is consistent. As already known from literature [36,37], all ion concentration changes were in a non-critical range and therefore the influence on cell proliferation and activity was quite low. To overcome a reduced Ca^2+^ content, the cement could be supplemented with Sr^2+^ ions triggering similar pathways in osteoblasts [38,39,40,41]. The overall Ca^2+^ adsorption and PO_4_^3−^ release is high, but the whole system around an implant must be considered. In vivo, the implant is surrounded by body fluids ensuring a steady removal of dissolution products and the supply with fresh nutrients. This would stabilize the ion concentration for cellular ingrowth.

## 3. Materials and Methods

### 3.1. Fiber Modification

C-fibers with an average length of 10 mm and a diameter of 7 µm were desized in boiling propan-2-ol followed by a chemical pre-functionalization with either hydrogen peroxide (H_2_O_2,_ 30 vol %, Carl Roth, Karlsruhe, Germany) or *aqua regia* for 40 min. *Aqua regia* was prepared by mixing 75 of 37 vol % hydrochloric acid (HCl, Carl Roth, Karlsruhe, Germany) and 25 of 65 vol % nitric acid (HNO_3_, Carl Roth, Karlsruhe, Germany). Finally, pretreated C-fibers were stirred in 1 M CaCl_2_ solution for 24 h at 80 °C, prepared by dissolving calcium hydride (CaH_2,_ Alfa Aesar, Karlsruhe, Germany) in ultra-pure water, followed by the dissolution of the resulting calcium hydroxide precipitate in HCl.

Physicochemical alterations of the fibers’ surfaces were characterized by using X-ray photoelectron spectroscopy (XPS, Quantum 2000, Physical Electronics, Chanhassen, MN, USA). Single peak fittings for both spectra, O1s and C1s, were performed with the software “fityk” using Gaussian equations. Contact angle analyses (Tensiometer DCAT21, Dataphysics, Filderstadt, Germany) of modified fibers were carried out in water. For this purpose, the surface tension of water *γ*_lv_ was estimated by the Wilhelmy plate method. Using the measured mass *m* of the advancing cycle and the fiber diameter *d*, which was estimated from SEM micrographs to be 6.7 µm, the contact angle *θ* was calculated according to Equation (1):

(1) $$\cos\theta =\frac{\Delta m\;g}{\pi \;d\;\gamma_{\mathrm{lv}}}$$

### 3.2. Cement Synthesis

α-tricalcium phosphate (α-TCP) was prepared by sintering calcium hydrogen phosphate (CaHPO_4_, purity ≥ 98%, Mallinckrodt-Baker, Griesheim, Germany) and calcium carbonate (CaCO_3_, purity ≥ 99%, Merck, Darmstadt, Germany) in a molar ratio of 2:1 for 5 h at 1400 °C followed by quenching to room temperature. The sintered cake was crushed and passed through a 125-μm sieve followed by milling in a planetary ball mill (PM400 Retsch, Haan, Germany) at 200 rpm for 4 h [42]. A solution of 1 M trisodium citrate (Na_3_C_6_H_5_O_7_, Carl Roth, Karlsruhe, Deutschland) and 2.5 wt % disodium hydrogen phosphate (Na_2_HPO_4_, Carl Roth, Karlsruhe, Deutschland) was added to the mixture of α-TCP and 1 to 3 wt % C-fibers with a powder to liquid ratio (PLR) of 3 g∙mL^−1^. For the initial setting of CDHA, the samples were exposed to 100% humidity for 4 h at 37 °C and thereafter transferred into distilled water for the final setting period of 7 days. Mixing α-TCP powder with an aqueous solution leads to the dissolution of TCP followed by the precipitation of calcium deficient hydroxyapatite (CDHA) (Equation (2)):

3 α Ca_3_(PO_4_)_2_ + H_2_O → Ca_9_(PO_4_)_5_(HPO_4_)OH (2)

Sodium dihydrogen phosphate (Na_2_HPO_4_) accelerates this reaction, reducing the initial setting time for a PLR of 3 g mL^−1^ to 25 min. Additionally, sodium citrate (Na_3_C_6_H_5_O_7_) was added to avoid particle agglomeration and pore formation in the set cement. This CPC system was reported in detail by Gbureck et al. [43,44]. The cement was characterized by X-ray diffraction (XRD, D5000, SIEMENS Diffractometer, Berlin, Germany) using CuK α radiation (*λ* = 0.15405 nm), 40 kV operation voltage, and 30 mA operating current. The scanning speed during measurement was 0.02°/s for the angular range of 25–55° 2*θ*. Scanning electron microscopy (SEM, Sigma VP, Carl-Zeiss, Oberkochen, Germany) of pure cement, reinforced cement, and fractured specimens was performed after 7 days of setting followed by drying. The kinetics of the setting reaction were characterized using a Gilmore needle test to estimate the initial (needle diameter 2.12 mm, weight 113 g) and final (needle diameter 1.06 mm, weight 454 g) setting time. Hereby, plate specimens with a diameter of 20 mm were used. 

Mechanical properties were characterized by three-point bending tests with a support span of *L* = 20 mm using a universal testing machine (Z020, Zwick, Ulm, Germany). The loading rate was 1 mm·min^−1^ and the preload was set to 0.1 N. The bending strength *σ*_b_ was calculated following Equation (3). The work of fracture WOF was estimated analogue to Xu et al. by using Equation (4) [19]. Hereby, the area between displacement *s*_0_ = 0 mm and the displacement at failure *s*_failure_ of the force *F* was calculated and normalized to the crosshead of the sample (width *b*, height *h*). However, due to the strong fiber orientation dependence on the residual strength at high strain, the error of the WOF until failure with ultimate strains up to 50% is vast. For a more expressive comparison, the WOF was calculated to the limit of 5% strain (WOF5), which is closer to the actual strain of bone [45] and therefore more meaningful for clinical applications as well. The WOF5 was calculated using only the area up to a strain of 5% (displacement *s*_5_), as proposed in Equation (5).

(3) $$\sigma_{b}=\;\frac{M_{b}}{W}=\;\frac{3FL}{2bh^{2}}$$

(4) $$WOF=\frac{\int_{s_{0}}^{s_{\mathrm{failure}}}Fds}{b\times h}$$

(5) $$WOF5=\;\frac{\int_{s_{0}}^{s_{5}}Fds}{b\times h}$$

### 3.3. In Vitro Biocompatibility

The biological behavior of fiber reinforced CPC was investigated by an elution test with the osteoblast-like cell line MG63. For this purpose, disks with a diameter of 15 mm and a height of 2 mm were used in 24-well plates. Samples (*n* = 6) were immersed in 1 mL cell culture media Dulbecco’s Modified Eagle’s Medium (DMEM-F12, life technologies, Carlsbad, CA, USA) supplemented with 1% penicillin/streptomycin (life technologies, Carlsbad, CA, USA) and 10% fetal calf serum (FCS, life technologies, Carlsbad, CA, USA). For dissolution, samples were stored at 37 °C and 5% CO_2_. Media was exchanged daily and transferred to cell culture. MG63 was seeded with 8.333∙10^3^ cells⋅mL^−1^ and cultured on cell culture plastic with *n* = 4 in 48-well plates. After a cultivation of 1 day, cells were exposed to the first dissolution media extracted from the samples. The day of the first exposure was defined as day 0. Fresh media served as reference. Cells were incubated at 37 °C and 5% CO_2_. Samples under investigation included CPC without fiber addition, CPC with 1% untreated C-fibers, and CPC with 1% C-fibers treated with *aqua regia* and CaCl_2_. After 3, 6, and 10 days, cell number and cell activity were determined. The latter was quantified with a water-soluble tetrazolium (WST-1) test. For this purpose, cells were incubated at 37 °C for 30 min with WST-1 reagent. The cell activity was analyzed with a spectrometer (Tecan, Männedorf, Switzerland) at a wavelength of 450 nm. For the calculation of cell numbers, cells were first detached by incubation with Accutase (PAA Laboratories GmbH, Pasching, Austria) for 10 min at 37 °C. Subsequently detachment was stopped by media addition. Cell counting was performed on a Casy counter (Roche Innovatis AG, Bielefeld, Germany) in isotone solution.

Additionally, the supernatant was investigated in regard to the ion release from dissolving samples. Inductively coupled mass spectrometry (ICP-MS, Varian, Darmstadt, Germany) was used with standard solutions of 100, 500, and 1000 ppb for calcium and phosphorus ions. The adsorption and release of ions was calculated with respect to the ion concentration in fresh media.

## 4. Conclusions

The mechanical properties of CPC in terms of strength and work of fracture could be significantly enhanced by incorporating chemically modified C-fibers. The fiber activation has a strong influence on the heterogeneous nucleation of calcium phosphate crystals on the fiber surface and, consequently, on the fiber–matrix interface. It was shown that besides crack deflection and fiber pull-out in case of a weak fiber–matrix interface, multiple matrix cracking occurred preferably when activated fibers were utilized. In vitro biocompatibility tests using the osteoblast-like cell line MG63, showed no inhibitory effects of the fiber–cement composite on cell proliferation and activity. Thus, these novel CPC composites might be of particular interest for the design of injectable and load-bearing bone substitutes.

## Figures and Tables

**Table 1:** Water contact angle of chemically treated fibers and setting kinetics of calcium phosphate cement (CPC) composites.

Chemical Treatment	Contact Angle (°)	Initial Setting Time (min)	Final Setting Time (min)
Pure CPC	-	25.0 ± 0.5	105 ± 5
untreated	71 ± 4	20.0 ± 0.5	65 ± 2
H_2_O_2_	64 ± 6	21.0 ± 0.5	80 ± 2
*aqua regia* followed by CaCl_2_	62 ± 4	18.5 ± 0.5	50 ± 2
